# ZmTE1 promotes plant height by regulating intercalary meristem formation and internode cell elongation in maize

**DOI:** 10.1111/pbi.13734

**Published:** 2021-11-09

**Authors:** Fengxia Wang, Zipeng Yu, Maolin Zhang, Mengli Wang, Xiaoduo Lu, Xia Liu, Yubin Li, Xiansheng Zhang, Bao‐cai Tan, Cuiling Li, Zhaojun Ding

**Affiliations:** ^1^ The Key Laboratory of Plant Development and Environmental Adaptation Biology Ministry of Education College of Life Sciences Shandong University Qingdao Shandong China; ^2^ School of Life Science Anhui Agricultural University Hefei Anhui China; ^3^ Maize Research Institute Shandong Academy of Agricultural Sciences/National Engineering Laboratory of Wheat and Maize/Key Laboratory of Biology and Genetic Improvement of Maize in Northern Yellow‐huai River Plain Ministry of Agriculture Jinan China; ^4^ College of Agronomy Qingdao Agricultural University Qingdao China; ^5^ State Key Laboratory of Crop Biology College of Life Sciences Shandong Agricultural University Tai'an China

**Keywords:** Maize, Cell division, Cell elongation, Auxin signalling, Nuclear‐cytoplasmic trafficking, Plant height, Phosphorylation, ZmTE1

## Abstract

Maize height is determined by the number of nodes and the length of internodes. Node number is driven by intercalary meristem formation and internode length by intercalary cell elongation, respectively. However, mechanisms regulating establishment of nodes and internode growth are unclear. We screened EMS‐induced maize mutants and identified a dwarf mutant *zm66*, linked to a single base change in *TERMINAL EAR 1* (*ZmTE1*). Detailed phenotypic analysis revealed that *zm66* (*zmte1‐2*) has shorter internodes and increased node numbers, caused by decreased cell elongation and disordered intercalary meristem formation, respectively. Transcriptome analysis showed that auxin signalling genes are also dysregulated in *zmte1‐2*, as are cell elongation and cell cycle‐related genes. This argues that ZmTE1 regulates auxin signalling, cell division, and cell elongation. We found that the ZmWEE1 kinase phosphorylates ZmTE1, thus confining it to the nucleus and probably reducing cell division. In contrast, the ZmPP2Ac‐2 phosphatase promotes dephosphorylation and cytoplasmic localization of ZmTE1, as well as cell division. Taken together, ZmTE1, a key regulator of plant height, is responsible for maintaining organized formation of internode meristems and rapid cell elongation. ZmWEE1 and ZmPP2Ac‐2 might balance ZmTE1 activity, controlling cell division and elongation to maintain normal maize growth.

## Introduction

Changes in plant height impact crop yield and thus food security. Dwarfism or semi‐dwarfism can be advantageous for crops as it can lead to increased lodging resistance, denser growth, and a higher harvest index, beneficial to production (Zhang *et al*., 2019). The vegetative shoot apical meristem (SAM) supports plant growth and development. The SAM contains a pool of undifferentiated cells that can generate primordia for structures that are above ground, including leaves. During vegetative growth, leaves initiated from leaf primordia follow a conserved distichous phyllotaxy. This means that new leaves initiate on the opposite side of the meristem compared to the previous leaf (Jackson and Veit, 1994). The maize stem consists of a phytomer unit, which includes leaves, leaf nodes, internodes, and axillary meristem units (Zhang, Sun, *et al*., 2018). Stem growth is largely due to increases in node number and the elongation of internode region. Both node cells and internode cells come from the same cell pool, namely the SAM (Tsuda *et al*., 2017).

In the last few years, several mutants that are defective in internode elongation have been isolated. Rice D50, a putative Inositol Polyphosphate 5‐Phosphatase (5PTase), has been reported to promote intercalary meristem (a meristem developing between regions of mature tissue) formation through regulating cell division direction, deposition of cell wall pectins, and actin organization (Sato‐Izawa *et al*., 2012). Os‐GRF1 (Oryza sativa‐GROWTH‐REGULATING FACTOR1) is involved in GA‐induced stem elongation (van der Knaap and Kim, 2000). In maize, several GA‐related mutants have also been isolated, including *dwarf1* (*d1*), *d3*, *d5*, *anther ear1* (*an1*), and the dominant mutants *D8* and *D9*. All these mutants affect internode elongation throughout the maize lifecycle. The narrow leaves and short internodes of the dwarf mutant *gif1* are associated with a reduction in undifferentiated cells in both leaves and stems (Zhang, Sun, *et al*., 2018). The shortened internodes and development‐deficient intercalary cells of *brevis plant1* (*bv1*) are both linked to deficient auxin transport (Avila *et al*., 2016). Similarly, the shortened lower internodes of *dwarf brachytic2* (*br2*) result from reduced auxin efflux out of the shoot and root meristem (Knoller *et al*., 2010; Zhang *et al*., 2019). BLH12/14 are KNOTTED1 (KN1) cofactors and maintain intercalary meristems and prevent precocious internode differentiation through interaction with KN1 (Tsuda *et al*., 2017).

Auxin, as an important phytohormone regulating cell division, cell elongation, and cell differentiation, plays roles in almost all stages of plant growth and development (Ma and Grones, 2018). Polar auxin transport, mediated by auxin efflux carriers such as PINFORMEDS (PINs) and auxin influx carriers such as AUXIN/LIKE AUXINs (AUX/LAXs), is involved in a various plant growth and development (Carraro *et al*., 2006). PIN1, as an auxin efflux carrier, is the main regulator of auxin distribution in the SAM (Carraro *et al*., 2006; de Reuille *et al*., 2006). Decreased expression of *ZmPIN1* at the incipient leaf primordium results in delayed leaf initiation, an enlarged SAM and altered phyllotaxis of *aberrant phyllotaxy1* (*abph1*, also known as *abphyl1*) (Lee *et al*., 2009). In addition, AUXIN SIGNALING FACTORs (ARFs) also promote cell expansion, cell division, and cell wall reconfiguration by up‐ or down‐regulating specific target genes (Chandler, 2016). The *Small Auxin Up RNA* (*SAUR*) gene family comprises plant‐specific cellular effectors that affect both auxin levels and auxin polar transport (Ren and Gray, 2015; Stortenbeker and Bemer, 2019). The majority of *SAUR* genes, including *AtSAUR10*, *AtSAUR19/63*, and *AtSAUR32/50,* are well documented as promoting *Arabidopsis* epicotyl growth by enhancing H^+^‐ATPase activity and cell elongation (Bemer *et al*., 2017; Spartz *et al*., 2014; Stortenbeker and Bemer, 2019).

Although several maize genes have been identified as regulators of intercalary meristem formation and internode cell expansion (Ballesteros *et al*., 2013; Tsuda *et al*., 2017; Zhang, Sun, *et al*., 2018), the underlying molecular mechanisms remain elusive. Therefore, we performed a high‐throughput screen of an EMS‐induced maize mutant library and identified a dwarf mutant *zm66* with shorter internodes and increased node numbers. Further investigations showed that the dwarf phenotype of *zm66* was caused by a mutation in *ZmTE1*, which has been shown to encode a key factor in maize height regulation (Veit *et al*., 1998). The poorly characterized ZmTE1 protein is highly similar to *Schizosaccharomyces pombe* Meiotic inducer 2 (Mei2), which encodes an RNA‐binding protein containing three conserved RNA recognition motifs (RRMs). Mei2 has been reported to promote meiosis in yeast cells by promoting premeiotic DNA synthesis (Watanabe and Yamamoto, 1994). In contrast, the Pat1 kinase phosphorylates Mei2, thereby inhibiting the transition from mitosis to meiosis (Watanabe *et al*., 1997). In this study, we identified a decisive role of ZmTE1 in the maintenance of intercalary meristem formation, internode cell elongation, and plant height regulation. ZmPP2Ac‐2 and ZmWEE1 may regulate phosphorylation and subcellular localization of ZmTE1, thus impacting cell division, cell elongation, and plant height.

## Results

### The *ZmTE1* is essential for maize plant height

**Figure 1:**
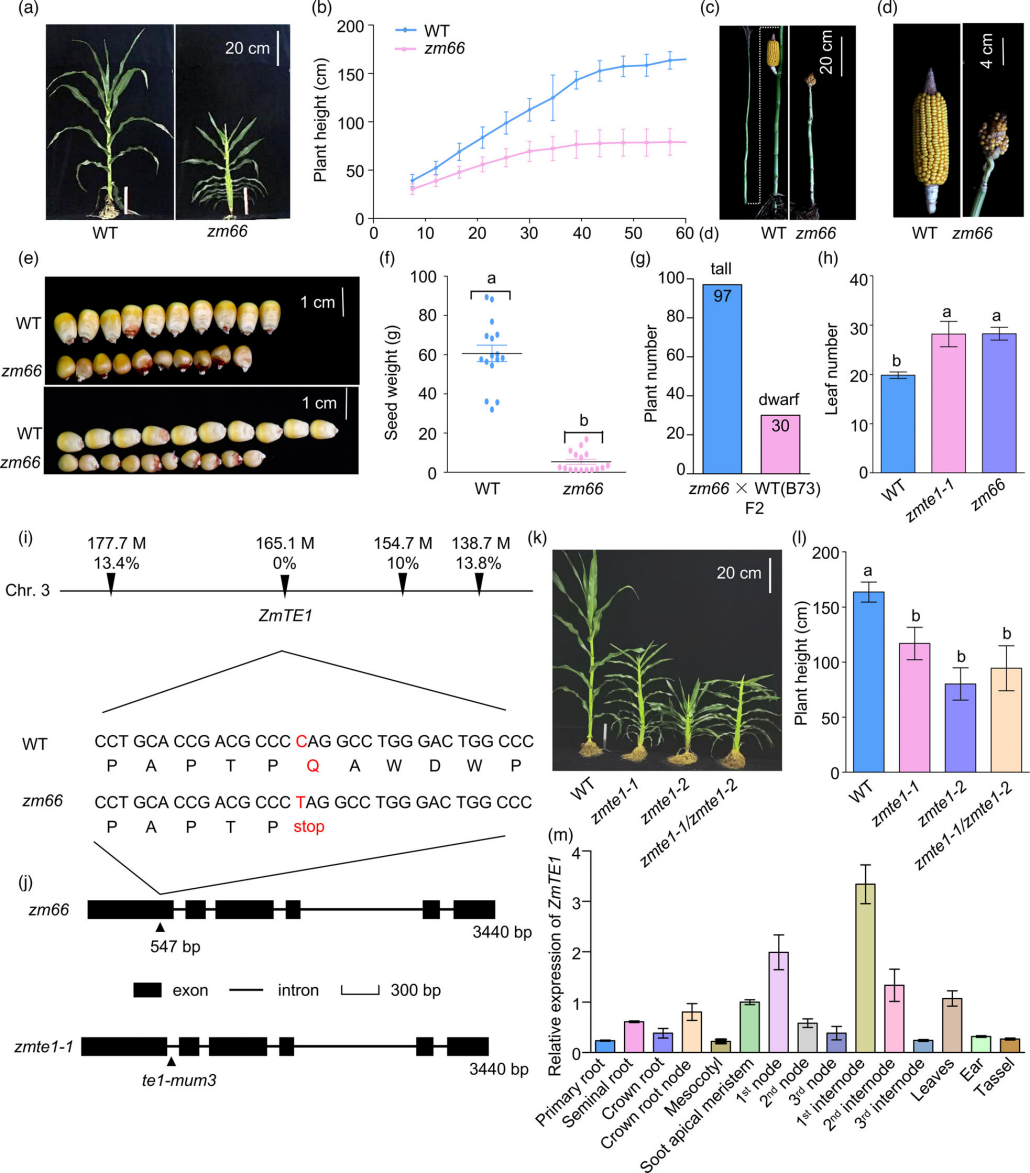
Isolating and mapping the *zm66* mutant. (a) *zm66* has reduced plant height phenotype during vegetative development. (b) Growth trend of *zm66* compared to WT. (c) The attachment mode of ear in mature WT and *zm66*. (d) The mature ear phenotype in WT and *zm66* mutant. (e) Seeds size of WT and *zm66*. (f) Mature seeds weight statistical in WT and *zm66*. Mean ± SD (one‐way ANOVA, *P* < 0.05; n ≥ 15). (g) Plant height phenotype in F2 generation plants from a *zm66* × WT cross (B73). (h) Leaf number in 60‐day‐old WT, *zmte1‐1*, and *zm66* plants. Data are shown as mean ± SE (one‐way ANOVA, *P* < 0.05; n ≥ 20). (i) Exome capture‐based MutMap analysis and mutation site analysis of *zm66* mutant. The percentage represents the linkage between the SNP mutation site and the coding gene controlling the *zm66* dwarf phenotype: The smaller the value, the stronger the linkage. Please refer to Materials and Methods for a more detailed description. (j) Schematic map of mutations in *zmte1‐1* and *zmte1‐2*. (k) Plant height phenotype in 60‐day‐old WT, *zmte1‐1*, *zmte1‐2*, and the F1 generation of *zmte1‐1/zmte1‐2* mutant plants. (l) Plant height statistical in 60‐day‐old WT, *zmte1‐1*, *zmte1‐2*, and F1 generation *zmte1‐1/zmte1‐2* plants. Mean ± SE (one‐way ANOVA, *P* < 0.05; n ≥ 20). (m) Relative expression of *ZmTE1* in the primary roots, seminal roots, crown roots, crown root nodes, mesocotyl and shoot apical meristem of two‐week‐old WT and in the nodes, internodes, leaves, ear, and tassel of six‐week‐old WT plants.

Maize height is determined by the number of nodes and the length of internodes, which are closely related to the formation of the intercalary meristem and the elongation of internode cells (Tsuda *et al*., 2017; Zhang *et al*., 2019; Zhang, Sun, *et al*., 2018). To elucidate the molecular mechanisms underlying intercalary meristem formation and internode cell elongation, we screened a maize EMS‐induced mutant library and identified a dwarf mutant, *zm66* (Figure 1a, b). Except for dwarfism, this mutant showed the tassel feminization further contributes to earlike appearance and seeds formation in place of the normal terminal tassel (Figure 1c, d), and the seeds size and weight per plant were seriously decreased (Figure 1e, f). The mutant was backcrossed to inbred B73 (hereafter WT) to remove phenotype‐independent changes. The F2 generation of *zm66* × B73 showed a phenotype segregation ratio of 3:1 (tall/short) in plant height (Figure 1g), indicating that the dwarfing phenotype of *zm66* is due to a single recessive gene. In order to identify the mutation site of *zm66*, we obtained 28,594 single nucleotide polymorphisms (SNPs; Excel S1) from 40 seedlings displaying mutant phenotypes from the *zm66* × B73 F2 population using the exome capture‐based MutMap (EcMutMap) analysis (Lu *et al*., 2018) (Figure 1i). After filtering, 10,687 transition mutations (i.e., G‐A or C‐T) were identified, including 315 SNPs with high‐quality scores (minor allele frequency ≥ 90%). Among them, four SNPs (SNP‐177676403, SNP‐165174753, SNP‐154716744, and SNP‐138739421) corresponding to non‐synonymous mutations in the coding regions on chromosome 3 were linked to the mutant and the recombination frequencies were 13.4%, 0.0%, 10.0%, and 13.8%, respectively (Figure 1i). The SNP‐165174753 was defined by the *ZmTE1* (GRMZM2G085113) gene, which had a 0% recombination frequency and is responsible for the dwarfing phenotype. A change of C‐to‐T at 547 bp in the first exon of *ZmTE1* changes a glutamine to a premature stop codon (Figure 1i, j). ZmTE1 was previously reported to play an essential role in preventing premature leaf initiation and development (Veit *et al*., 1998), a phenotype consistent with the increased leaf number in the *zm66* mutant (Figure 1h). Therefore, we renamed *zm66* as *zmte1‐2* following *zmte1‐1* identified previously (Veit *et al*., 1998). We crossed the *zmte1‐1* allele with *zmte1‐2* (Figure 1k). The F1 plants of *zmte1‐1/zmte1‐2* also showed a dwarf phenotype similar to *zmte1‐1* and *zmte1‐2* (Figure 1k, l), suggesting that plant‐height phenotype of *zmte1‐2* is caused by loss of *ZmTE1* function.

ZmTE1, an RNA binding protein, contains three RNA recognition motifs (RRM1, RRM2, and RRM3) and shows a high similarity to *Schizosaccharomyces pombe* Mei2, *Oryza sativa* LHD2/PLA2/OML1, and *Arabidopsis thaliana* AtTEL1/AtTEL2 (Jeffares *et al*., 2004; Kawakatsu *et al*., 2006). To better understand the evolutionary relationship of Mei2‐like proteins, we generated a phylogenetic tree based on Mei2‐like proteins from *Schizosaccharomyces pombe*, *Zea mays*, *Oryza sativa*, and *Arabidopsis thaliana*. We uncovered nine Mei2‐like proteins in *Zea mays*, seven in *Oryza sativa*, and eight in *Arabidopsis thaliana* (Figure S1). Although there are nine Mei2‐like proteins in maize, loss of *ZmTE1* causes a dwarf phenotype (Figure 1a, b), implying an essential role in plant growth regulation. *ZmTE1* is expressed in almost all maize tissues (Figure 1m), suggesting its functional importance in diverse tissues. Notably, *ZmTE1* expression was relatively high in both internodes and leaves (Figure 1m), further indicating that the developmental abnormalities in both leaves and stems of *zmte1‐2* plants are caused by the functional loss of *ZmTE1*.

### ZmTE1 promotes internode elongation by accelerating cell elongation

**Figure 2:**
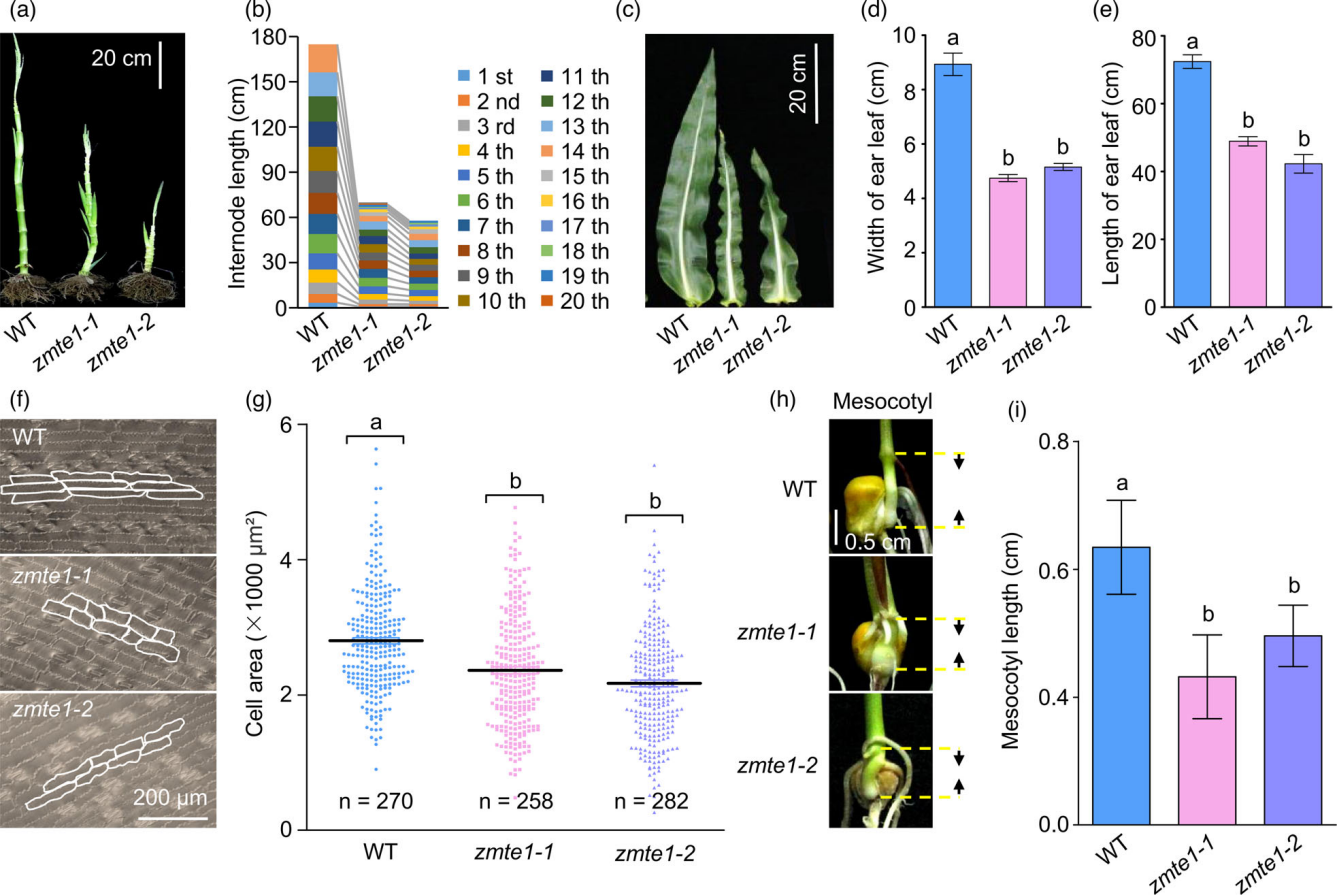
ZmTE1 promotes internode cell elongation. (a and b) Stem phenotype and internode length in 60‐day‐old WT, *zmte1‐1*, and *zmte1‐2* plants. (c) Ear leaf phenotype in 60‐day‐old WT, *zmte1‐1*, and *zmte1‐2* plants. (d and e) Maximum width and length of ear leaves in 60‐day‐old WT, *zmte1‐1*, and *zmte1‐2* plants. Mean ± SE (one‐way ANOVA, *P* < 0.05; n ≥ 20). (f) Lower epidermal cell phenotype of leaves in 60‐day‐old WT, *zmte1‐1*, and *zmte1‐2* plants. (g) Quantification of lower epidermal cell area. Mean ± SD (one‐way ANOVA, *P* < 0.05; n ≥ 258). (h and i) The mesocotyl phenotype and mesocotyl length of two‐week‐old WT, *zmte1‐1*, and *zmte1‐2* plants. Mean ± SE (one‐way ANOVA, *P* < 0.05; n ≥ 20).

Plant height decreased by 33% and 50%, in mature *zmte1‐1* and *zmte1‐2* plants, respectively (Figure 1l). We found that *zmte1* mutants have shortened internodes, accounting for reduced plant height (Figure 2a, b). All internodes of *zmte1‐1* and *zmte1‐2* are shorter than those of WT, with the 10th, 11th, 12th, and 13th internodes particularly affected (Figure 2b; Figure S2). In addition, other important agronomic traits, such as leaf number, leaf size, and internode length, were also altered in *zmte1* mutants compared to WT (Figures 1h, 2a‐c). The maximum width and length of ear leaves were particularly affected, being reduced by 47%, 36% and 32%, 46%, respectively (Figure 2c‐e).

The previous work indicates that internode length mainly depends on internode cell elongation (Tsuda *et al*., 2017), suggesting that ZmTE1 may positively regulate internode cell elongation. Unfortunately, due to the high degree of fibrosis in the nodes, we could not obtain good stem tissue sections and therefore could not directly examine the length of intercalary cells. Since *zmte1‐1* and *zmte1‐2* mutant leaves were also significantly smaller than WT (Figure 2c‐e), we examined cell morphology of the lower epidermal cells (Figure 2f) and found that these cells were smaller in the *zmte1‐1* and *zmte1‐2* mutants compared to WT (Figure 2f, g). The mesocotyl, located between the root and stem, is largely responsible for pushing shoots out of the soil (Saab and Ho, 1995), and its growth is similar to that of the *Arabidopsis* hypocotyl, i.e. linked to cell elongation (Kutschera and Wang, 2016). Mesocotyl development in *zmte1‐1* and *zmte1‐2* plants also visibly lagged behind that of WT (Figure 2h, i), further confirming that ZmTE1 promotes cell elongation. Considering that leaf shrinkage and mesocotyl shortening of *zmte1‐1* and *zmte1‐2* are both caused by limited cell elongation, and internode elongation is related to cell elongation (Tsuda *et al*., 2017), we hypothesize that ZmTE1 increases internode length by promoting internode cell elongation.

### ZmTE1 maintains intercalary meristem formation and cell division

**Figure 3:**
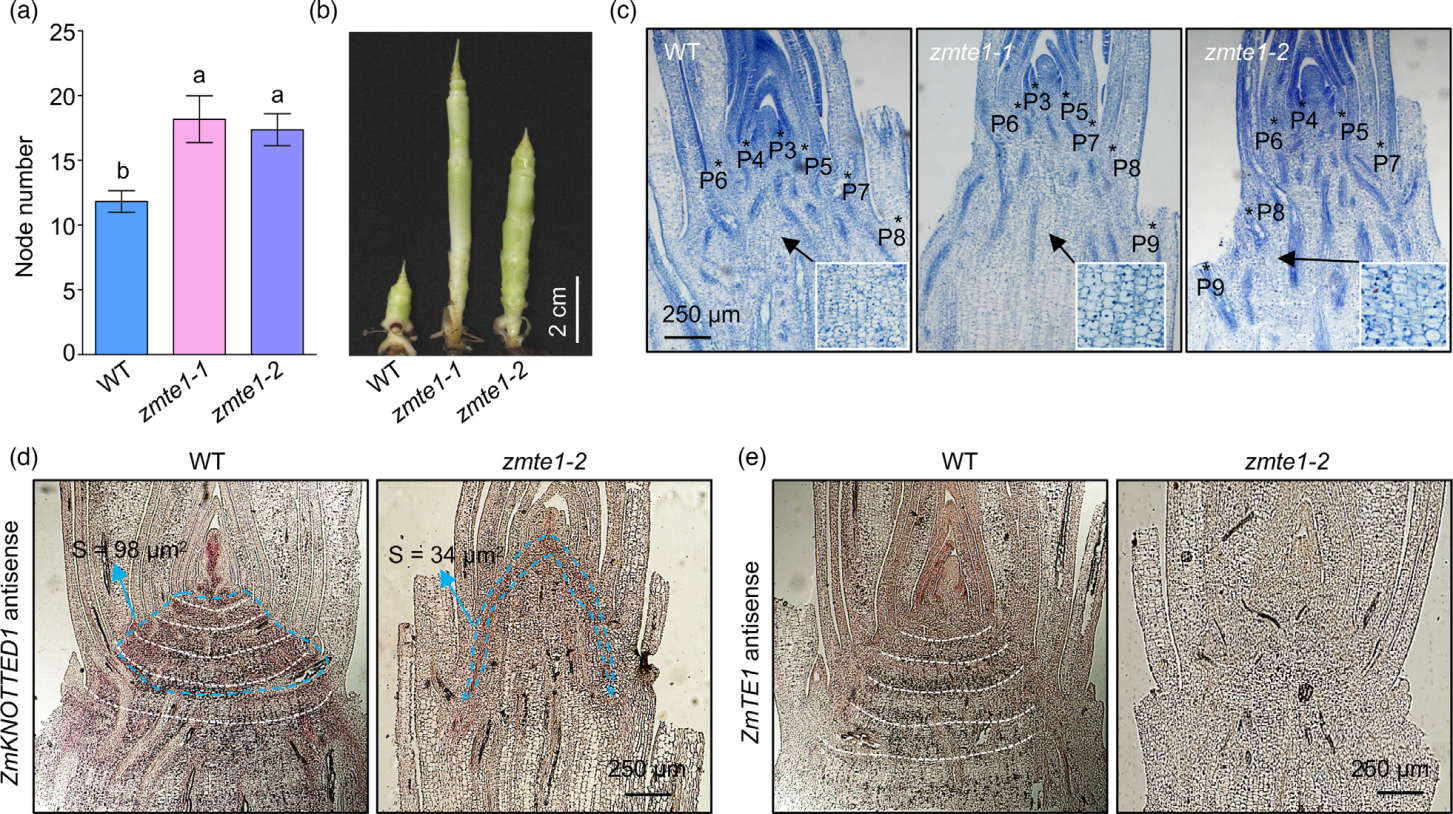
ZmTE1 positively regulates intercalary meristem formation and cell division. (a) Quantification of node number in 60‐day‐old WT, *zmte1‐1*, and *zmte1‐2* plants. Data are shown as mean ± SE (one‐way ANOVA, *P* < 0.05; n ≥ 20). (b) The stem phenotype of four‐week‐old WT, *zmte1‐1*, and *zmte1‐2* mutants. (c) Stem tissue sections from 20‐day‐old WT, *zmte1‐1*, and *zmte1‐2* plants. P1‐P9 represent sites of initiation of leaf primordia or leaves. (d and e) Expression of *ZmKOTTED1* and *ZmTE1* viewed by RNA *in situ* hybridization in the stem four‐week‐old WT and *zmte1‐2*. The blue dotted line represents the boundary of meristems, and the white dotted line represents the hierarchical structure of intercalary meristems.

In addition to internode length, the number of nodes also determines plant stem height (Zhang, Sun, *et al*., 2018). Surprisingly, the dwarf mutants *zmte1‐1* and *zmte1‐2* had 50% more nodes than WT (Figures 2a, 3a), implying that ZmTE1 maybe also have an important role in node formation regulation. Moreover, the number of nodes in *zmte1‐1* and *zmte1‐2* mutant plants increased significantly at the seedling stage (Figure 3b). This result suggests that increased nodes present at the mature stage are due to accelerated node formation at the seedling stage. Based on tissue section analysis of the stem from 20‐day‐old plants, we found that initial internode elongation appeared between the eighth and ninth leaf in *zmte1‐1* and *zmte1‐2* plants. This timing is earlier than that of WT where internode elongation begins between the seventh and eighth leaf (Figure 3c). Therefore, accelerated node formation alleviated the slowing stem growth resulting from decreased cell elongation during early growth of *zmte1‐1* and *zmte1‐2* mutants.

*KN1*, specifically expressed in the SAM and intercalary meristem (Tsuda *et al*., 2017), is commonly used to determine the location and area of the meristem. However, the results of RNA *in situ* hybridization showed that the meristem area of *zmte1‐2* was significantly smaller than that of WT (Figure 3d). Our data suggest that ZmTE1 promotes cell division at the meristem, since the size of the meristem is mainly due to the number of cells (Zhang, Sun, *et al*., 2018). Furthermore, the regular hierarchical structure in the WT stem gradually develops into intercalary meristems (Tsuda *et al*., 2017). In contrast, *zmte1‐2* stem had a disordered structure (Figure 3d, e), suggesting that ZmTE1 also plays a critical role in the ordered formation of the intercalary meristem. Therefore, loss of ZmTE1 regulation in *zmte1‐2* plants results in an arbitrary formation of intercalary meristems and subsequent over‐formation of nodes (Figure 3a). Taken together, ZmTE1 plays a dual role in promoting cell division in the meristem and in maintaining intercalary meristem formation for full growth potential.

### ZmTE1 positively regulates auxin signalling

**Figure 4:**
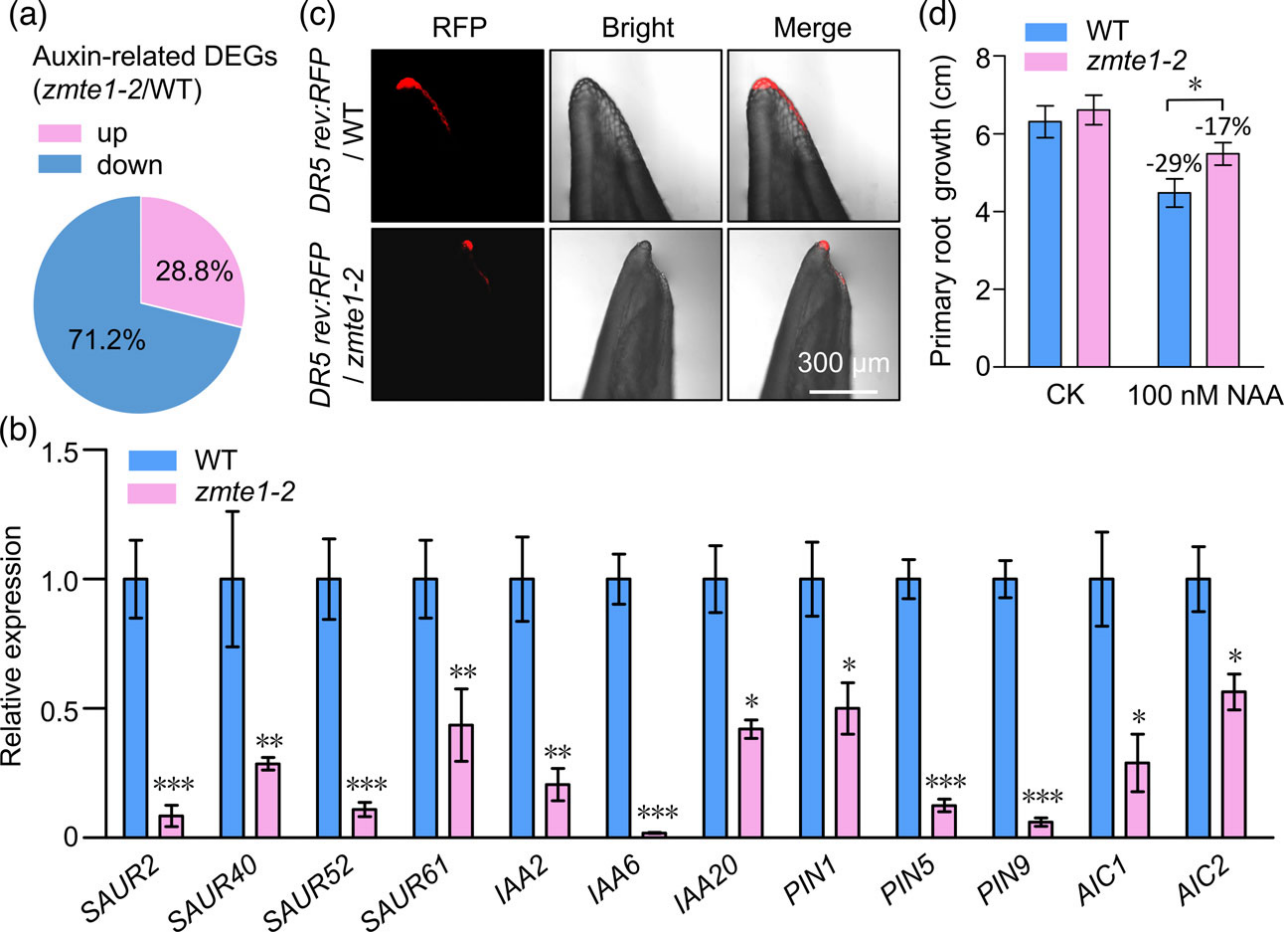
ZmTE1 maintains auxin signalling. (a) Auxin‐related DEGs (*zmte1‐2*/WT) from RNA‐seq analysis. (b) qRT to measure the relative expression of auxin signalling‐related genes, including *SAURs*, *Aux/IAAs*, *PINs*, and *AICs*. Data are shown as mean ± SE (Student’s *t* test, *P ≤ 0.05; **P ≤ 0.01; ***P ≤ 0.001). (c) RFP signal in the SAM of two‐week‐old *DR5rev:RFP*/WT and *DR5rev:RFP*/*zmte1‐2* plants. (d) Primary root growth in WT and *zmte1‐2* mutant treated with or without 100 nM NAA for 48 h. Data are shown as mean ± SE (Student’s *t* test, *P ≤ 0.05; n ≥ 15).

To deepen our understanding of *ZmTE1*‐mediated cell elongation and cell division, we performed RNA sequencing (RNA‐seq) on nodes as well as internodes from 28‐day‐old WT and *zmte1‐2* plants. 5,546 differentially expressed genes (DEGs; *P‐value* ≤ 0.05 and fold change ≥ 2) were found in *zmte1‐2* nodes compared to WT (Excel S2). Kyoto Encyclopedia of Genes and Genomes (KEGG) analysis revealed that 200 auxin signalling‐related genes are present in the DEG dataset (Figure S3). Given the important role of auxin in regulating cell elongation and cell division (Du and Spalding, 2020), the down‐regulated auxin cluster genes in *zmte1‐2* (Figure 4a) might contribute to developmental phenotypes in these mutants.

*Aux/IAAs* and *SAURs* are auxin rapidly induced genes (Abel and Theologis, 1996) which are usually used as marker genes for the auxin signalling response. In subsequent detailed qRT validation experiments, all these genes were significantly down‐regulated in *zmte1‐2* (Figure 4b), confirming a decrease in auxin signalling response in *zmte1‐2*. More directly, we introduced the maize *DR5 rev:RFP* (auxin response marker) to *zmte1‐2* to visually observe auxin signalling intensity at SAM. The results showed that RFP fluorescence intensity was significantly decreased in *zmte1‐2* (Figure 4c), suggesting that ZmTE1 plays an important role in the maintenance of essential auxin signalling at SAM. To further confirm the positive regulation of auxin signalling by ZmTE1, we verified whether inhibition of root elongation by high concentrations of auxin was weakened in *zmte1‐2*. Consistent with our hypothesis, auxin‐mediated inhibition of root elongation was significantly reduced when *ZmTE1* function was lost (Figure 4d), reinforcing the important role of *ZmTE1* in auxin signalling. It is also worth mentioning that some auxin transporter genes were also significantly reduced in *zmte1‐2* mutant plants (Figure 4b), implying that ZmTE1 may enhance auxin signalling by regulating auxin transport. Specifically, *PIN1* has been reported to play an important role in regulating auxin distribution during SAM development (Carraro *et al*., 2006). Down‐regulation of *PIN1* expression in *zmte1‐2* suggests that the abnormal development of the SAM may be due to disruption of auxin distribution and insufficient auxin signalling (Figure 4b). There was no significant difference in the expression of auxin synthesis genes between WT and *zmte1‐2* (Excel S2), indicating that ZmTE1‐mediated regulation of auxin signalling could be independent of auxin synthesis. In summary, ZmTE1 positively regulates auxin signalling.

### ZmTE1 regulates the expression of cell division‐ and cell elongation‐related genes

To further investigate the molecular mechanism underlying cell division and cell elongation defects in *zmte1‐2*, we carefully analysed the RNA‐seq data again. A number of cell cycle‐related genes, including *CYCAs*, *CYCBs*, and *CDKs*, were significantly down‐regulated in *zmte1‐2* (Figure S4a). This result suggests that normal meristem cell division requires the maintenance of ZmTE1, which is consistent with meristem developmental defects observed in *zmte1‐2* (Figure 3d). Consistent with cell elongation defects in *zmte1‐2*, genes linked to cell elongation, including *SAURs*, *EXPANSIONs*, and *EXTs*, also showed significantly decreased expression in *zmte1‐2* (Figure 4b; Figure S4b). In addition, some genes that negatively regulate cell elongation, such as *PEROXIDASEs* (*PERs*) (Knoller *et al*., 2010), were significantly up‐regulated (Figure S4b). These results indicate that ZmTE1 is a necessary maintenance factor for cell elongation, consistent with the observed shortening of internodes and mesocotyls in *zmte1‐2* (Figure 2a, h). It has been well documented that auxin can promote cell elongation by up‐regulating the expression of *SAURs*, *EXPANSIONs*, and *EXTs* (Du *et al*., 2020), and down‐regulating the expression of *PERs* (Knoller *et al*., 2010), supporting the idea that auxin signalling is essential for ZmTE1‐induced cell elongation. In summary, ZmTE1 maintains meristem formation and internode elongation through auxin‐mediated regulation of cell division and cell elongation.

### ZmTE1 interacts with ZmPP2Ac‐2 and ZmWEE1

**Figure 5:**
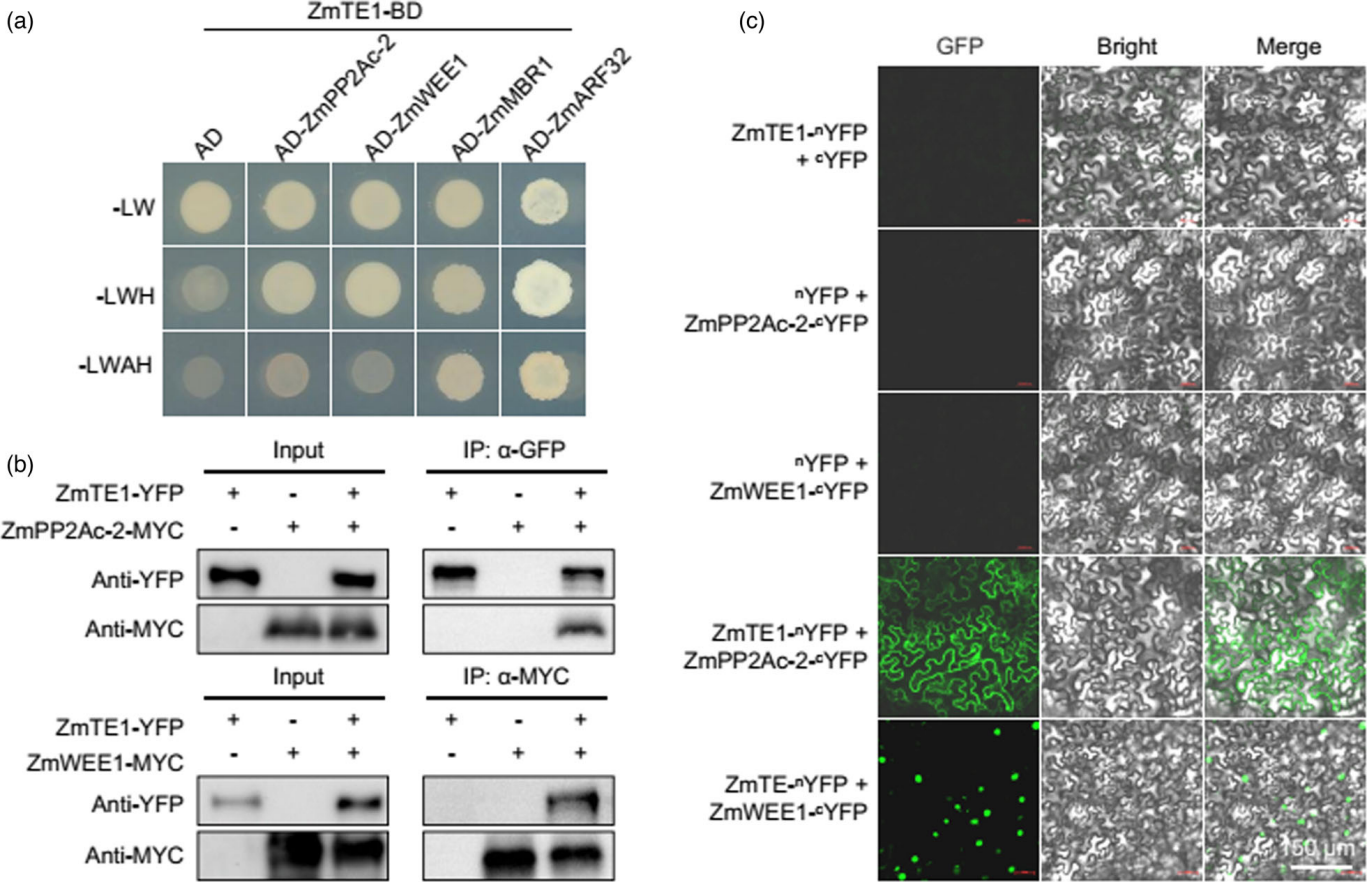
ZmTE1 interacts with ZmPP2Ac‐2 and ZmWEE1. (a) ZmTE1 interacts with ZmPP2Ac‐2, ZmWEE1, ZmMBR1, and ZmARF32 in yeast. (b) Co‐IP assays in *Arabidopsis* mesophyll protoplasts verify the interaction between ZmTE1 and ZmWEE1 or ZmPP2Ac‐2. (c) BiFC assay performed in *N. benthamiana* leaves to verify the interaction between ZmTE1 and ZmWEE1 or ZmPP2Ac‐2.

To further elucidate how ZmTE1 regulates cell elongation, cell division, and intercalary meristem formation, we used ZmTE1 as a bait protein to screen a maize cDNA library. We identified four proteins as ZmTE1 interacting partners, ZmMBR1, ZmARF32, ZmWEE1, and ZmPP2Ac‐2 (Figure 5a). AtMBR1 (GRMZM2G165044, designated ZmMBR1 in this study), an E3 ligase homolog AtMED25‐BINDING RING‐H2 PROTEIN1, has been reported to promote flowering (Inigo *et al*., 2012). ZmARF32 is homologous to the transcription factor AtARF17, as a ZmTE1 binding partner. AtARF17 has been reported to play a key role in anther dehiscence and pollen wall pattern formation (Xu *et al*., 2019). Neither of these proteins are likely be involved in internode cell elongation or cell division at the meristem. ZmWEE1 is a homolog of the WEE1 kinase which acts as a cell cycle G2/M check point to inhibit cell cycle operation in response to DNA replication stress (Velappan and Signorelli, 2017) and is one of the potential binding partners of ZmTE1. In addition, ZmPP2Ac‐2 was also found to interact with ZmTE1 based on yeast two‐hybrid analysis. ZmPP2Ac‐2 is homologous to the phosphatases AtPP2A‐C3 and AtPP2A‐C4 which promote auxin polar transport by increasing PIN1 polar localization through dephosphorylation. These phosphatases have been reported to regulate cell elongation and meristem formation (Ballesteros *et al*., 2013). We also confirmed the interaction between ZmTE1 and ZmWEE1 and ZmPP2Ac‐2 using bimolecular fluorescence complementation (BiFC) and co‐immunoprecipitation (CoIP) assays in tobacco leaf epidermal cells (Figure 5b, c).

### ZmWEE1 and ZmPP2Ac‐2 regulate phosphorylation status and subcellular localization of ZmTE1

**Figure 6:**
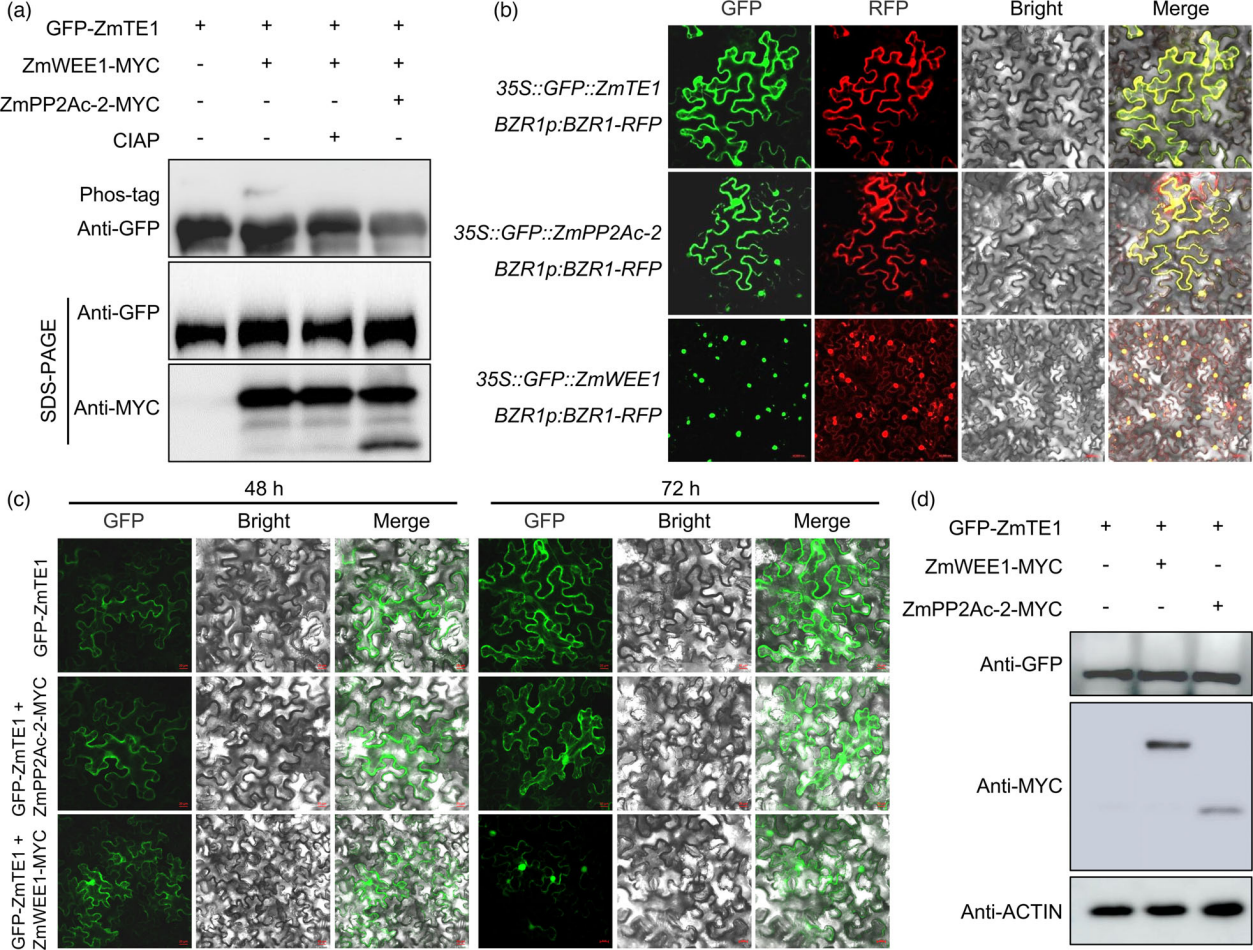
ZmWEE1 and ZmPP2Ac‐2 control the subcellular localization of ZmTE1 through phosphorylation status. (a) ZmTE1 and ZmPP2Ac‐2 phosphorylate and dephosphorylate ZmTE1, as seen using *in vivo* phosphorylation assays in *Arabidopsis* mesophyll protoplasts. (b) Subcellular localization analysis of ZmTE1, ZmWEE1, and ZmPP2Ac‐2 performed in *N. benthamiana* leaves. *BZR1p:BZR1‐RFP* indicates cytoplasmic vs. nuclear localization. (c) Localization of ZmTE1 is regulated by ZmWEE1 and ZmPP2Ac‐2 in a co‐transformation assay in *N. benthamiana* leaves. (d) Co‐transformation assay performed in *N. benthamiana* leaves detects whether the protein stability of ZmTE1 is regulated by ZmWEE1 and ZmPP2Ac‐2.

Since ZmWEE1 kinase and ZmPP2Ac‐2 phosphatase both interact with ZmTE1, we speculate that ZmWEE1 and ZmPP2Ac‐2 might affect the function of ZmTE1 through regulating phosphorylation and dephosphorylation, respectively. To test this hypothesis, we conducted *in vivo* phosphorylation experiments in *Arabidopsis* protoplasts. Co‐transformation of ZmWEE1 with ZmTE1 resulted in the appearance of an additional protein band, which was removed upon calf intestinal alkaline phosphatase treatment (CIAP) (Figure 6a), arguing that this protein was phosphorylated ZmTE1. Notably, ZmWEE1‐mediated phosphorylation of ZmTE1 was also eliminated by ZmPP2Ac‐2 (Figure 6a), indicating that ZmTE1 can be phosphorylated and dephosphorylated by ZmWEE1 and ZmPP2Ac‐2, respectively. These data confirm that these factors have opposing effects on ZmTE1 phosphorylation and may tune ZmTE1 function through opposing regulation of this post translational modification.

Phosphorylation modifications can affect protein stability, activity, subcellular localization, or interaction with other proteins (Yu *et al*., 2019; Zhang *et al*., 2021). Given that the fluorescence of ZmTE1‐ZmPP2Ac‐2 was observed in both cytoplasm and nucleus, while the fluorescence of ZmTE1‐ZmWEE1 was specifically observed in the nucleus (Figure 5c), we speculate that ZmTE1 phosphorylation alters the subcellular localization of ZmTE1. In the subsequent subcellular localization analysis, both ZmTE1 and ZmPP2Ac‐2 were found to localize to the cytoplasm and nucleus, while ZmWEE1 was specifically localized in the nucleus (Figure 6b). However, in the co‐transformation experiments, ZmWEE1 almost completely confined ZmTE1 to the nucleus (Figure 6c), implying that phosphorylated ZmTE1 prefers to be localized to the nucleus. Consistent with this hypothesis, in the presence of ZmPP2Ac‐2, the de‐phosphorylated ZmTE1 tended to localize to the cytoplasm (Figure 6c). Furthermore, co‐transformation of ZmWEE1‐ZmTE1 or ZmPP2Ac‐2‐ZmTE1 did not affect protein levels of ZmTE1 (Figure 6d), indicating that ZmTE1 phosphorylation primarily affects its subcellular localization rather than protein stability. Considering the consistent roles of ZmPP2Ac‐2 and ZmTE1 in regulating auxin transport, cell elongation, and meristem formation (Awotunde *et al*., 2000; Ballesteros *et al*., 2013; Yue *et al*., 2016), as well as the opposite roles of ZmWEE1 and ZmTE1 in regulating the cell cycle (Velappan *et al*., 2017), ZmPP2Ac‐2‐mediated dephosphorylation of ZmTE1 and nuclear export might promote auxin signalling, cell division, cell elongation, and meristem formation, while ZmWEE1 may inhibit ZmTE1 function.

## Discussion

Plant height impacts lodging and is therefore pivotal in determining crop yield. This is especially true for maize and rice, where height depends on the number of nodes and the length of internodes (Tsuda *et al*., 2017; Zhang, Sun, *et al*., 2018). To elucidate the molecular mechanism underlying intercalary meristem formation and intercalary cell elongation, we screened an EMS‐induced maize mutant library and identified a dwarf mutant *zm66* with shorter internodes and increased node numbers. The dwarf phenotype of *zm66* was characterized as being caused by a mutation in *ZmTE1*, which is known to encode a key factor in maize height regulation (Veit *et al*., 1998). Our studies further show that ZmTE1 regulates maize height through the regulation of auxin signalling, cell division, cell elongation, and intercalary meristem formation.

Since ZmPP2Ac‐2, like ZmTE1, regulates auxin signalling, cell elongation, and meristem formation (Awotunde *et al*., 2000; Ballesteros *et al*., 2013; Yue *et al*., 2016), we examined the role of this phosphatase, and the opposing kinase, ZmWEE1 (Velappan *et al*., 2017). Our data argue that ZmPP2Ac‐2 and ZmWEE1 may have positive versus negative effects on ZmTE1 function through direct protein interactions. In addition, ZmWEE1 restricts ZmTE1 specifically to the nucleus, while ZmPP2Ac‐2 and ZmTE1 promote cytoplasmic localization (Figure 6b, c). Nuclear vs. cytoplasmic localization of ZmTE1 may therefore be key to regulating its function. By regulating this subcellular localization, ZmPP2Ac‐2 and ZmWEE1 may regulate the ability of ZmTE1 to promote cell division, cell elongation, and meristem formation. Cell division and cell elongation are necessary for plant growth, which means that shuttling of ZmTE1 from the cytoplasm to the nucleus may occur under normal conditions. However, since ZmWEE1 is linked to shutting down cell division during DNA damage to allow time for DNA repair (Velappan *et al*., 2017), ZmWEE1‐mediated inhibition of ZmTE1‐mediated cell cycle may therefore be especially important during DNA damage. Upon DNA damage repair and recovery of plant growth, this subcellular restriction will also be relieved, as ZmTE1 phosphorylation is erased by ZmPP2Ac‐2 (Figure 6a).

Mmi1 is an RNA‐binding protein crucial for the removal of hazardous meiosis‐specific mRNAs during the mitotic cell cycle (Yamanaka *et al*., 2010). It has been reported that Mei2, a yeast homologue of ZmTE1, can suspend mitosis and initiate meiosis by inhibiting Mmi1 function after entering the nucleus from cytoplasm (Mukherjee and Futcher, 2018). This argues that Mei2 in the nucleus reduces cell division. If nuclear ZmTE1 in maize inhibits cell division, expression of relevant genes should be significantly increased in *zmte1‐2*, which is not the case (Figure S4a). We therefore hypothesize that transfer of ZmTE1 (which contains an RNA recognition motif) to the cytoplasm does not remove inhibition of genes related to cell division, but instead transfers the mRNAs of these genes to the cytoplasm to accelerate their translation. Unfortunately, limited by the low sensitivity of methods for mRNA detection, we were unable to isolate ZmTE1‐bound mRNAs from ZmTE1‐GFP proteins expressed in *Arabidopsis* protoplasts.

Yeast two‐hybrid experiments argue that ZmTE1 interacts with seven ZmARFs members (Figure 5A and S5), which are core transcription factors linked to auxin signalling (Chandler, 2016). Considering the significant decrease in auxin signalling caused by loss of *ZmTE1* function (Figure 4a‐c), ZmTE1 may positively regulate auxin signalling. Therefore, the interaction between ZmTE1 and ZmARFs seems to enhance the expression of auxin responsive genes, a specific mechanism that remains open for exploration in future studies.

**Figure 7:**
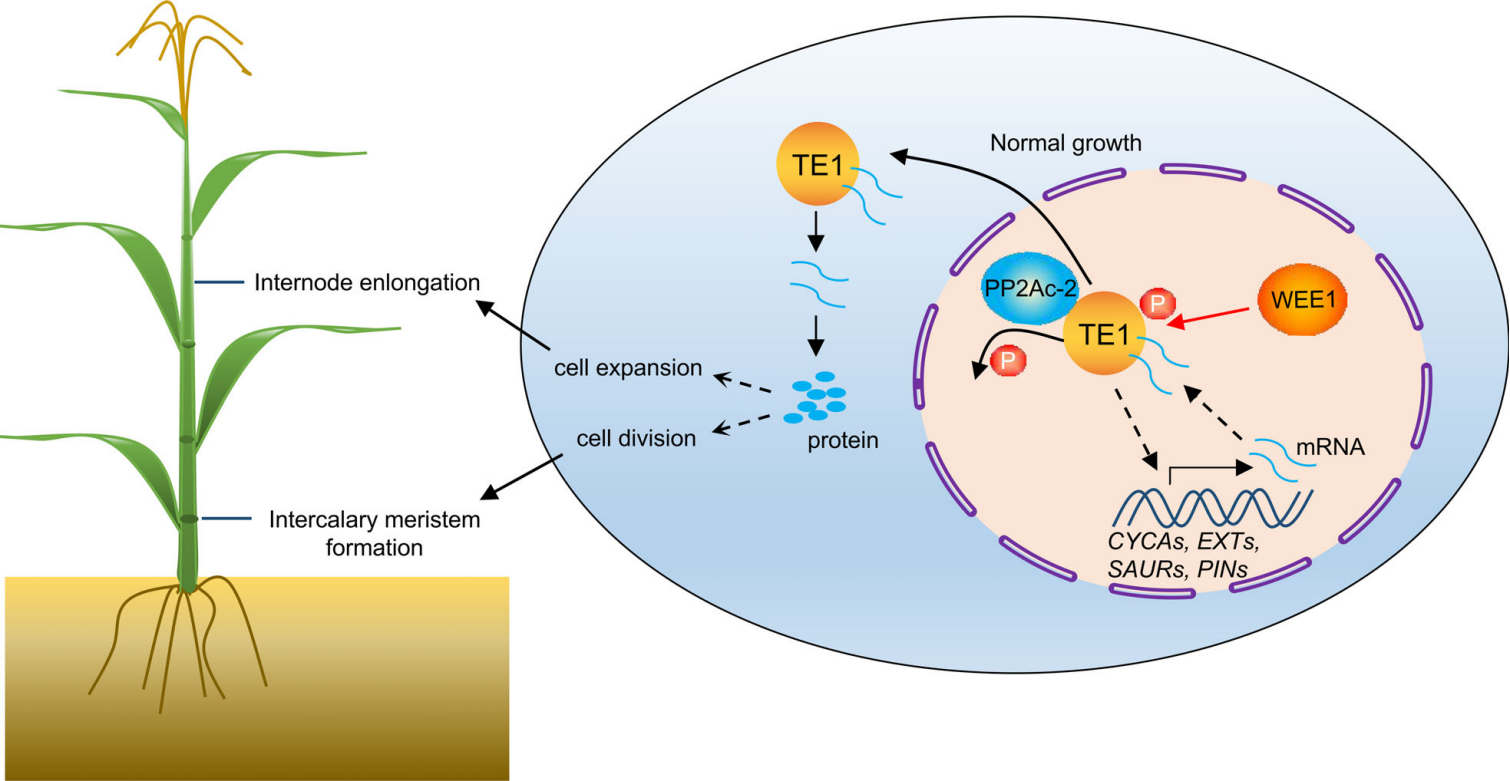
ZmWEE1 and ZmPP2Ac‐2 regulate ZmTE1 function through phosphorylation. Schematic model for ZmTE1 regulation of plant height. Under normal conditions, ZmTE1 promotes the expression of cell division‐ and cell elongation‐related genes, thus increasing levels of these mRNAs in the nucleus. In contrast, ZmPP2Ac‐2 maintains transfer of ZmTE1 from the nucleus to cytoplasm through dephosphorylation, which may bring bound mRNAs to the cytoplasm, thus increasing protein levels from these transcripts. Vigorous cell elongation and cell division accelerate internode elongation and normal internode meristem formation, respectively, which maintain normal plant growth. However, ZmWEE1 phosphorylated ZmTE1 restricts it to the nucleus, resulting in the restriction of the normal processes of cell division and cell elongation.

In summary, a working model has been proposed to illustrate how ZmTE1 promotes plant height by regulating intercalary meristem formation and internode cell elongation in maize (Figure 7). ZmTE1 might indirectly control the transcription of genes related to cell cycle, cell elongation, and auxin signalling, thereby increasing the concentration of these mRNAs in the nucleus. ZmPP2Ac‐2 probably maintains the transfer of ZmTE1 from the nucleus to the cytoplasm through dephosphorylation, which simultaneously brings these bound mRNAs into the cytoplasm and enhances the translation of these mRNAs into functional proteins. On the contrary, ZmWEE1 phosphorylates ZmTE1 and restricts these bound mRNAs to the nucleus. However, the exact direct target mRNAs of ZmTE1 and the fine‐tuning balance of ZmTE1 activity by ZmWEE1 and ZmPP2Ac‐2 remain to be further investigated in the future.

## Materials and methods

### Dwarf mutant screening and *ZmTE1* gene identification

About 12 000 EMS‐induced maize mutants obtained from (Lu *et al*., 2018) were screened for phenotypes, including plant height, in the field. A dwarf mutant *zm66* (*zmte1‐2*) was isolated and backcrossed with wild‐type B73. F1 plants were self‐pollinated, and F2 plants were grown in the field, where plant height was measured in 80‐day‐old plants. We crossed *zmte1‐2* and *zmte1‐1* from the mutant library (https://www.maizegdb.org/) to check for allelism, based on the phenotype of F1 plants. Exome capture‐based MutMap (EcMutMap) (Lu *et al*., 2018) is an improved method of MutMap that is a way of rapid gene identification in rice through crossing mutants to WT (Abe *et al*., 2012; Takagi *et al*., 2013). In brief, the DNA of 40 F2 seedlings showing the dwarf phenotype was extracted using the CTAB method and was mixed in an equimolar fashion, which was then subjected to EcMutMap by a GAIIx device (Illumina). Filtered reads were mapped onto the WT reference sequence using MAQ software (Li and Ruan, 2008). Polymorphisms were validated by sequencing the amplicons derived from each of the 40 templates and calculating the cross‐over rate (Zhang, Lu, *et al*., 2018). Where the variant site was associated with a pair of G/A or C/T peaks, crossing‐over in one of the two homologs was inferred; a single peak corresponding to G or C was interpreted as the occurrence of crossing‐over in both homologs, while a single A or T peak suggested that no crossing‐over had occurred. The crossing‐over rate (G/G+A or C/C+T) of each mutant was calculated. Mutants unlinked to the dwarf phenotype or located on different chromosomes were expected to segregate 1:1; those fully linked to the causative locus were not expected to segregate with respect to the phenotype. Variants at a position on the same chromosome (chromosome 3 in this study) linked to the causative locus were expected to be partially linked. The allelic status of *ZmTE1* was obtained by resequencing in each of the mutants, and the primers used for this assay are listed in Table S1.

### Response of primary root growth to exogenous NAA treatment

WT and *zmte1‐2* seeds were surface‐sterilized with 5% (W/V) NaClO for 30 min and washed with sterile water three times. After that, the seeds were grown hydroponically in a 28 °C/25 °C (day/night) chamber at ~60% relative humidity under a 16‐h‐light/8‐h‐dark photoperiod (~100 µmol m^‐2^ s^−1^). The culture solution, 0.5 × Hoagland liquid solution [0.51 g/L KNO_3_, 0.82 g/L Ca(NO_3_)_2_, 0.49 g/L MgSO_4_⋅7H_2_O, 0.136 g/L KH_2_PO_4_, 0.6 mg/L FeSO_4_, 2.86 mg/L H_3_BO_3_, 1.81 mg/L MnCl_2_⋅4H_2_O, 0.08 mg/L CuSO_4_⋅5H_2_O, 0.22 mg/L ZnSO_4_⋅7H_2_O, and 0.09 mg/L H_2_MoO_4_⋅4H_2_O], was renewed every two days. The three‐day‐old seedlings with primary root lengths of approximately 5 cm were treated with 100 nm NAA for 48 h.

### Phylogenetic analysis

To build a phylogenetic tree, Mei2‐like proteins in *Schizosaccharomyces pombe, Zea mays*, *Oryza sativa,* and *Arabidopsis* were aligned by ClustalX (Yu *et al*., 2020). The phylogenetic tree was built using this alignment output based on a neighbour‐joining method in MEGA7.

### Phenotype analysis and cytological observation

The phenotypes of *zmte1* mutants were analysed in detail, recording plant height, the number and length of nodes, the size and number of leaves, the length of the mesocotyl, and the size and weight of seeds. To measure cell size, mature leaves at the ear position of three individuals were sampled from *zmte1‐1*, *zmte1‐2*, and WT, respectively. The lower epidermal cells on the central region of the leaf were observed using an Olympus BX53 microscope. Three fields were observed for each leaf, and ~30 cells per field were measured. The average length of measured cells from five leaves was used to represent cell size for each genotype, and cell size was calculated using Image J software.

### RNA‐Seq, qRT‐PCR, and tissue expression pattern analysis

Three nodes from the last brace root of four‐week‐old WT and *zmte1‐2* mutant were collected for RNA isolation using TRIzol reagent (Invitrogen, Carlsbad, CA, USA). The purified RNA was sent to BGI (Shenzhen, China) for RNA‐seq analysis. Differentially expressed genes were identified using the thresholds *P‐value* ≤ 0.05 and |log^2^| ≥ 1. To visualize the auxin‐signalling response in the SAM, a maize transgene marker line carrying *DR5 rev:RFP* (Gallavotti *et al*., 2008) was crossed with the *zmte1‐2* mutant, and two‐week‐old seedlings of *DR5 rev:RFP/zmte1‐2* and *DR5 rev:RFP/*WT were scanned for the strength of RFP signal.

To analyse the expression of *ZmTE1* in different maize tissues, two‐week‐old primary roots, seminal roots, crown roots, crown root internode, mesocotyl, SAM, and six‐week‐old brace roots, three nodes and internodes, leaves, tassel, and ear were collected from WT plants. The RNAs from different tissues were extracted using the TRIzol reagent, and the expression of *ZmTE1* in different tissues was analysed using the qRT‐PCR assay. For qRT‐PCR, RNA was isolated from the nodes of WT and *zmte1‐2* and reverse‐transcribed using a Transcriptor First Strand cDNA Synthesis kit (Roche, Basel, Switzerland), following the manufacturer’s protocol. qRT‐PCR was performed in a MyiQTM Real‐time PCR Detection System (Bio‐Rad, Hercules, CA, USA) using ChamQ SYBR Color qPCR Master Mix (Q411, Vazyme, Nanjing, China). The *ZmACTIN* and *ZmGAPDH* genes were used as the reference controls. Primers used for qRT‐PCR are listed in Table S1.

### Paraffin section and *in situ* hybridization

SAM tissue from 14‐day‐old *zmte1* and WT plants was fixed in a solution containing 4% paraformaldehyde at 4 °C for 16 h, then washed twice with 1× PBS, and dehydrated through an ethanol series, that was then substituted with xylene, embedded in Paraplast Plus (Sigma‐Aldrich), and sectioned to a thickness of 8 mm. Plant tissue sections were stained with toluidine blue for histological observation, and the slides were imaged using an Olympus BX53 microscope.

*In situ* hybridization was performed as previously described (Zhou *et al*., 2011). In brief, we constructed sense and antisense RNA probes as follows. Primer sets for *ZmTE1* and *ZmKN1* were used to amplify 461 bp and 570 bp fragments, which were then cloned into *pSPT18* vector and linearized with *Hind*III and *EcoR*I, respectively. Sense and antisense probes were then synthesized using SP6 and T7 RNA polymerase, respectively, with Digoxigenin (Digoxigenin‐11‐UTP, Roche Diagnostics) as a label. Finally, standard paraffin sections were prepared and *in situ* hybridization performed as in a previous study (Zhou *et al*., 2011).

### Yeast two‐hybrid screen of cDNA library and protein interaction validation

Total RNA was extracted from 14‐day‐old WT seedlings, and genomic DNA was removed after DNaseI treatment. cDNA was synthesized using the SMART cDNA Library Construction Kit (Clontech) and sent to Takara (Osaka, Japan) to construct the cDNA library. The yeast two‐hybrid assay was performed according to the manufacturer’s manual and the Matchmaker GAL4 Two‐Hybrid System 3 (Takara, Osaka, Japan). The *CDS* of *ZmTE1* were cloned into the bait plasmid *pGBKT7* (BD), and yeast‐two‐hybrid screening was conducted according to the manufacturer’s instructions. To confirm the identified protein–protein interactions, the CDS of candidate genes were cloned into the *pGADT7* (AD). BD‐ZmTE1 and the AD‐candidate fusions (empty vector was used as negative control) were transferred together into yeast Y2HGold using the PEG/LiAc method. After culturing on synthetic medium plates (SD medium) lacking Trp and Leu (‐LW) for two days, the transformants were transferred onto SD‐Trp‐Leu‐His (‐LWH) and SD‐Trp‐Leu‐His‐Asp (‐LWAH) for an additional three or four days. Primers used in this assay are listed in Table S1.

### Co‐immunoprecipitation

The *ZmWEE1*, *ZmPP2Ac‐2*, and *ZmTE1* genes were cloned into the *pCAMBIA1390‐7Myc‐6HIS* or *pEareyGate 101‐YFP* vectors to generate the *35S::ZmTE1‐Myc*, *35S::ZmTE1‐YFP*, *35S::ZmPP2Ac‐2‐Myc*, and *35S::ZmWEE1‐YFP* plasmid, respectively. Constructs were then transformed into *Arabidopsis* mesophyll cells for transient protein expression. Co‐immunoprecipitation (Co‐IP) was performed according to a previous study (Lv *et al*., 2020). In brief, *Arabidopsis* mesophyll cells were harvested and lysed in cell lysis buffer (0.5 mm EDTA; 10 mm Tris‐HCl; pH 7.5; 0.5% NP‐40; 1 mm PMSF; 150 mm NaCl) on ice for 30 minutes with pipetting every 10 minutes. Cell lysates were centrifuged, and the supernatant was incubated with MYC‐Trap magnetic agarose beads (Chromotek, catalog number ytma20, Germany) at 4 °C for 2 h. The beads were washed three times with dilution buffer (10 mm Tris‐HCl; pH 7.5; 150 mm NaCl; 0.5 mm EDTA) and then re‐suspended in SDS loading buffer. The re‐suspended beads were boiled for 10 minutes followed by western blotting using anti‐MYC (Abclonal, catalog number AE010, Wuhan, China) or anti‐GFP (TransGen Biotech, catalog number HT801‐02, Beijing, China) antibody.

### Bimolecular fluorescence complementation and subcellular localization analysis

To generate the constructs for the bimolecular fluorescence complementation (BiFC) assays, full‐length CDS of *ZmPP2Ac‐2*, *ZmWEE1*, and *ZmTE1* were amplified and cloned into the *p2YN* and *2YC* vector for fusion with the N‐terminus and C‐terminus of YFP by linearizing with *Pac*I and *Asc*I, respectively. The plasmids were introduced into the *Agrobacterium* strain GV3101, which was injected into four‐week‐old *N. benthamiana* leaves with MMA medium (50 mm MES, 10 mm MgCl_2_, 20 µM acetosyringone, pH 5.6) for transient protein expression (Yu *et al*., 2019). The tobacco epidermal cells were then imaged by Confocal Laser Scanning Microscopy LSM880 (Zeiss, Germany) at 488 nm. Three biological repetitions were analysed for each combination, and the combination with empty vector was used as the negative control. For subcellular localization analysis, we cloned the CDS of *ZmPP2Ac‐2*, *ZmWEE1*, and *ZmTE1* into *pB7WGF2* vector using the LR reaction to generate the *35S::GFP‐ZmPP2Ac‐2*, *35S::GFP‐ZmWEE1*, and *35S::GFP‐ZmTE1* constructs, followed by transformation into GV3101. Then, GV3101 carrying *35S::GFP‐ZmPP2Ac‐2*, *35S::GFP‐ZmWEE1*, or *35S::GFP‐ZmTE1* and GV3101 harbouring *BZR1p:BZR1‐RFP* (used as nucleus and cytoplasm localization markers) were transformed together into *N. benthamiana* leaves. Fluorescence was assessed using the Zeiss LSM 880 confocal microscope (Zeiss, Germany).

### Phosphorylation and dephosphorylation assays *in vivo*

To identify whether ZmWEE1 and ZmPP2Ac‐2 affect the phosphorylation state of ZmTE1, GFP‐ZmTE1, ZmWEE1‐MYC, and ZmPP2Ac‐2‐MYC were transformed into *Arabidopsis* mesophyll protoplasts. The protoplasts were harvested and lysed in cell lysis buffer (0.5 mm EDTA; 10 mm Tris‐HCl; pH 7.5; 0.5% NP‐40; 1 mm PMSF; 150 mm NaCl) on ice for 30 min with pipetting every 10 min. Cell lysates were centrifuged, and the supernatant of ZmTE1 together with ZmWEE1 was treated with calf intestinal alkaline phosphatase (CIAP) at 30℃ for 30 min. At last, the supernatants were boiled for 10 min at 99 °C. The phosphorylated and dephosphorylated TE1 was separated and detected using phos‐tag (Yu *et al*., 2019). Anti‐GFP antibody (TransGen Biotech, catalog number HT801‐02) detected the protein level and phosphorylation of ZmTE1; the protein level of ZmWEE1‐MYC and ZmPP2Ac‐2‐MYC was detected using anti‐MYC antibody (Abclonal, catalog number AE010).

### Statistical analysis

Statistical analysis was performed using the Student’s *t* test (*P < 0.05, **P < 0.01, and ***P < 0.001) or one‐way ANOVA (*P* < 0.05; LSD and Duncan test). All experiments were repeated at least three times, and the data are shown as mean ± standard error (SE).

## Accession number

The RNA‐seq data are available in the Gene Expression Omnibus database under accession number GSE181794.

## Conflict of interest

The authors declare that they have no conflict of interest.

## Author contributions

Z.D. and C.L. conceived research plan; F.W. and M.W. performed experiments; X.D.L. assisted in experiments; F.W., Z.Y., and M.Z. analysed the data and made the figures; X.Z., Y.L., B.T., and X.L. provided some important suggestions; and F.W., Z.Y., C.L., and Z.D. wrote the manuscript. All authors read and approved the final manuscript.

## Supporting information


**Figure S1**. Phylogenetic analysis of ZmTE1
**Figure S2**. Node length analysis of the zmte1‐1 and zmte1‐2
**Figure S3**. KEGG analysis based on the DEGs from RNA‐seq
**Figure S4**. ZmTE1 regulates the expression of cell division‐ and cell elongation‐related genes
**Figure S5**. ZmTE1 interacts with ZmARFs in Y2H assay
**Table S1**. The primers used in this study
**Excel S1.** Variable nucleotides identified from the exome capture‐based sequencing assay
**Excel S2.** The DEGs in RNA‐seq
