# Supplementary material for: ZmTE1 promotes plant height by regulating intercalary meristem formation and internode cell elongation in maize
Source: Plant Biotechnol J. 2021 Nov 9;20(3):526–37. doi: 10.1111/pbi.13734 (PMC8882779; doi:10.1111/pbi.13734)
Supplement: Supplementary file 1 — Figure S1. Phylogenetic analysis of ZmTE1 Figure S2. Node length analysis of the zmte1‐1 and zmte1‐2 Figure S3. KEGG analysis based on the DEGs from RNA‐seq Figure S4. ZmTE1 regulates the expression of cell division‐ and cell elongation‐related genes Figure S5. ZmTE1 interacts with ZmARFs in Y2H assay Table S1. The primers used in this study Excel S1. Variable nucleotides identified from the exome capture‐based sequencing assay Excel S2. The DEGs in RNA‐seq [file PBI-20-526-s001.zip › pbi13734-sup-0001-SupInfo.pdf]

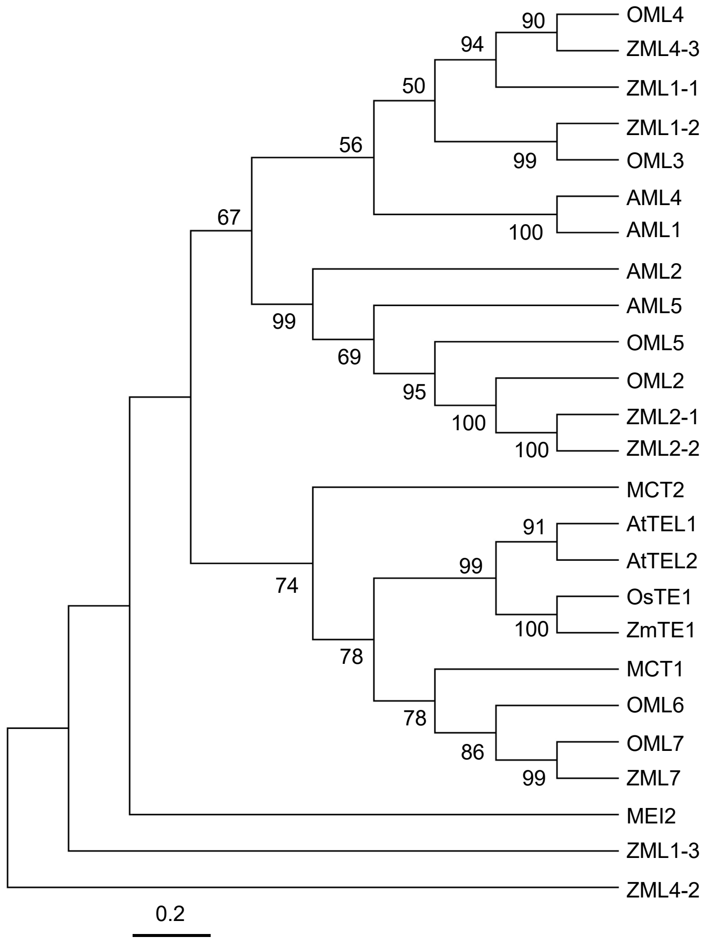

Figure S1. Phylogenetic analysis of *ZmTE1*

Phylogenetic tree analysis of Mei2-like proteins in maize, rice and *Arabidopsis* showed the similarity and homology relationship between ZmTE1 and the Mei2-like proteins.

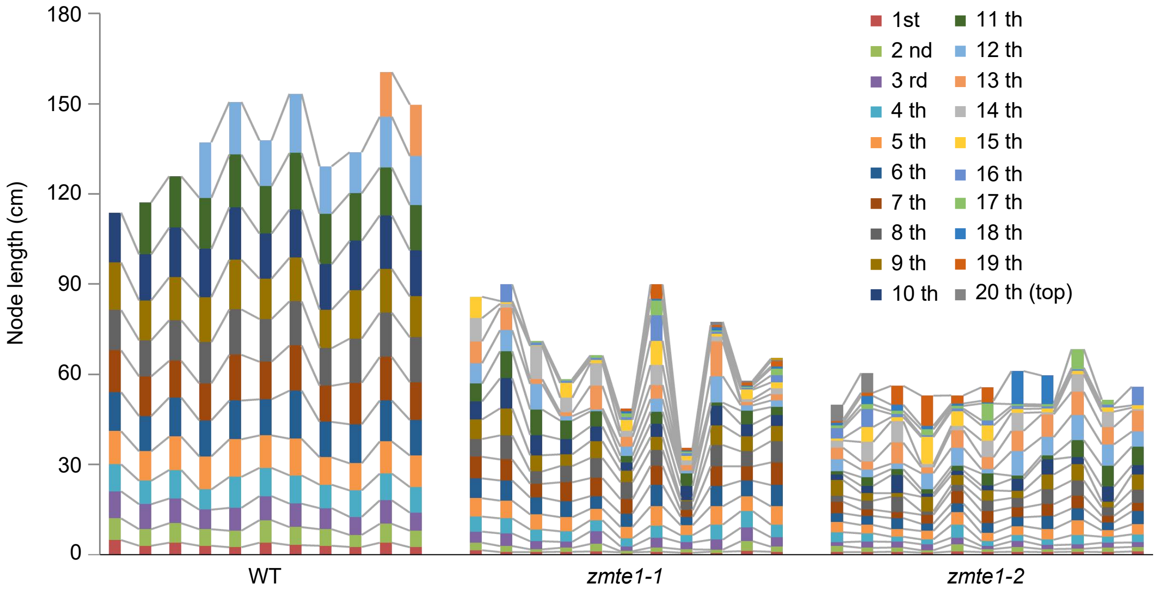

Figure S2. Node length analysis of the *zmte1-1* and *zmte1-2*

The analysis of the length of all the nodes in 11 separate individuals not only reflected that the length of the nodes in *zmte1-1* and *zmte1-2* mutants was shortened, but also reflected that the shortening occurred randomly at different nodes.

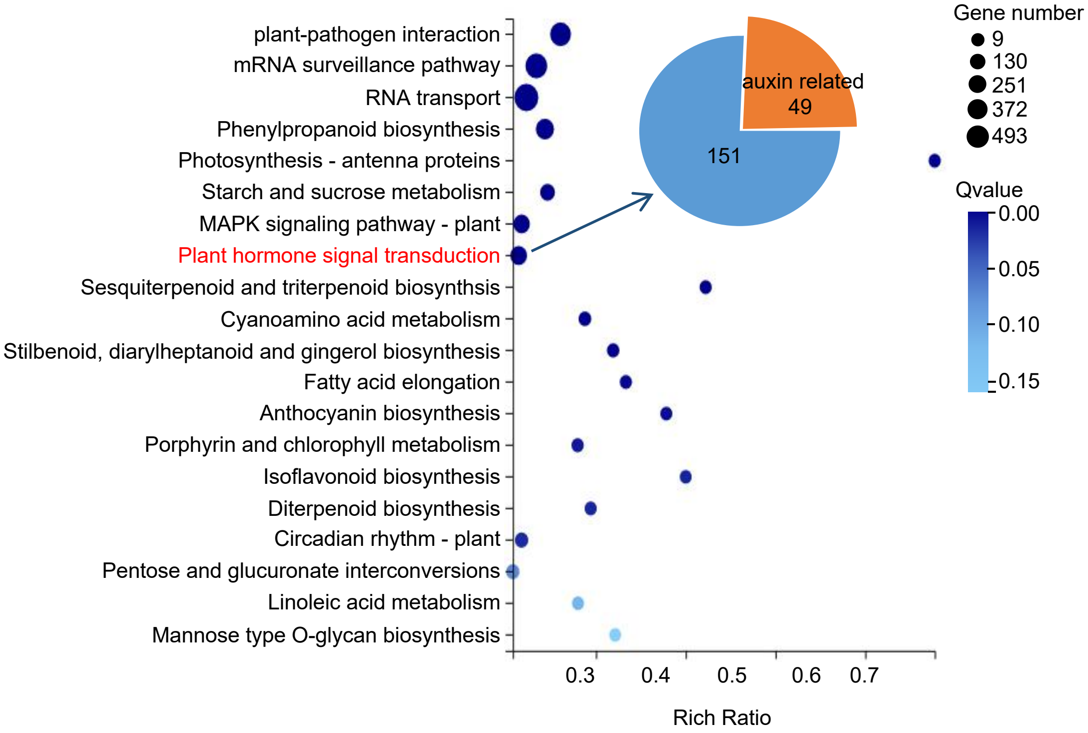

Figure S3. KEGG analysis based on the DEGs from RNA-seq

Among 5,546 DEGs, the “Plant hormone signal transduction” related gene cluster contained 200 members, among which 49 auxin related members were included.

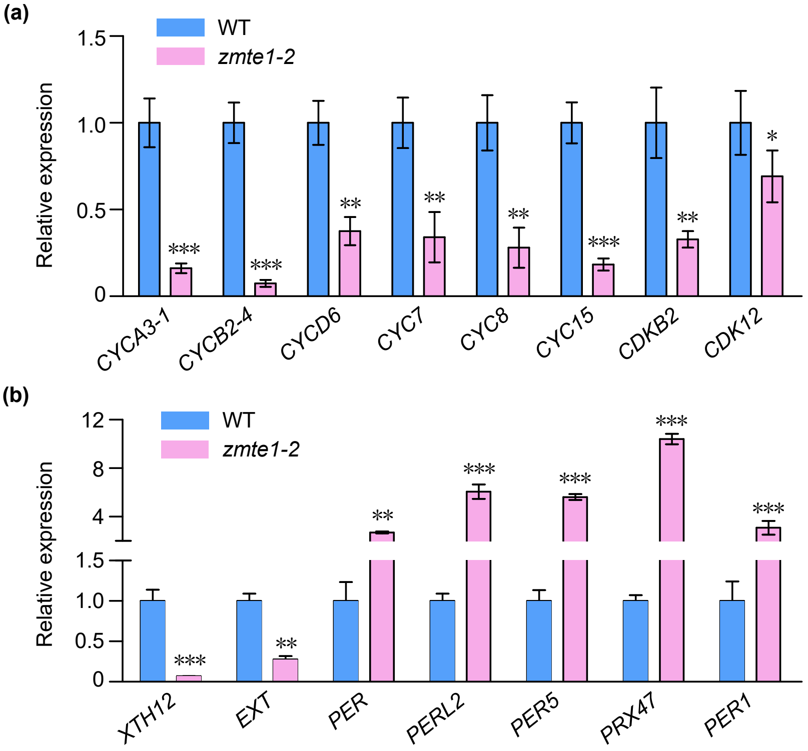

Figure S4. ZmTE1 regulates the expression of cell division and cell elongation related genes

(a) The expression of cell cycle related genes verified in qRT assay.

(b) The expression of cell expansion related genes verified in qRT assay.

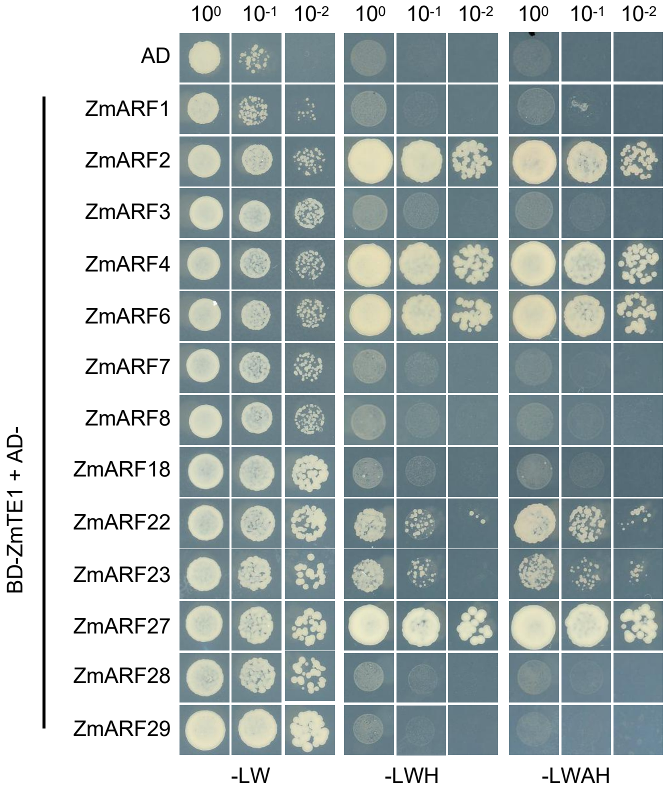

Figure S5. ZmTE1 interacts with ZmARFs in Y2H assay

The interation between ZmTE1 and the ZmARF family members.

Table S1. Primers used in this study

Purpose	Gene ID	Name	Sequence (5'-3')
Yeast Two-Hybrid Assay	GRMZM2G085113	ZmTE1-F	ATGGAGGGTGGGGGAGGGAG
	GRMZM2G085113	ZmTE1-R	GTCGTCGTAGCCAAGCCG
	GRMZM5G878541	ZmWEE1-F	ATGCCCCCGCCGCCGCCG
	GRMZM5G878541	ZmWEE1-R	ATGCCCCCGCCGCCGCCG
	GRMZM2G108355	ZmPP2A-F	ATGAGCAGCCCGCATGGC
	GRMZM2G108355	ZmPP2A-R	GACACCTGACTATTTCCTGTGA
	GRMZM2G165044	ZmMBR1-F	ATGGCTCGAAATAATGAGGGCC
	GRMZM2G165044	ZmMBR1-R	TGCAAAGCGGCCGCGGCC
	GRMZM2G169820	ZmARF1-F	ATGGAGGCGCCGGGGACGAGCTC
	GRMZM2G169820	ZmARF1-R	GAGGGTCACCGGCGACTAGAAA
	GRMZM2G153233	ZmARF2-F	ATGAAGGAGGTGGCCGAGGAA
	GRMZM2G153233	ZmARF2-R	AGCCATGTACTCGACGAGAGG
	GRMZM2G078274	ZmARF3-F	ATGAGCTCCTCGTCCGCGGCCAGCA
	GRMZM2G078274	ZmARF3-R	AGATAGGTAGCGTGGATCGTTTCC
	GRMZM2G034840	ZmARF4-F	ATGATGACGTCCTCGTACGAG
	GRMZM2G034840	ZmARF4-R	AGCCATTTGCATGCAGTCGTT
	GRMZM2G122614	ZmARF6-F	ATGCCCCCATCGCTGGAGCC
	GRMZM2G122614	ZmARF6-R	TTCAACTATGTGTGGATCCTT
	GRMZM2G475263	ZmARF7-F	ATGGCGCAAGGAGCAGGAC
	GRMZM2G475263	ZmARF7-R	GGATGGGAAGAGGGACTAT
	GRMZM2G352159	ZmARF8-F	ATGGCGGCGGCTGCCTGCG
	GRMZM2G352159	ZmARF8-R	GGCCTTCTTGCAGCTTCTG
	GRMZM2G035405	ZmARF18-F	ATGTGGCTTCGAGATACCGC
	GRMZM2G035405	ZmARF18-R	TCCCAACTCGACCGAACCGA
	GRMZM2G089640	ZmARF22-F	ATGTCTCATTGCCTTGTCAGGA
	GRMZM2G089640	ZmARF22-R	CTGCCAAGGGTCGTCGCCCA
	GRMZM2G441325	ZmARF23-F	ATGGGGATTGATCTCAACGCC
	GRMZM2G441325	ZmARF23-R	TATTCCCAAGGGAGCAGCACA
	GRMZM2G160005	ZmARF27-F	CGTCGCTCTCGCTGCCCCT
	GRMZM2G160005	ZmARF27-R	GGCCCTCCATGCATTTGCGT
	GRMZM2G006042	ZmARF28-F	ATGGTGGCGGCTGCAGCG
	GRMZM2G006042	ZmARF28-R	CTGACTGGACAAGTTAGCG
	GRMZM2G086949	ZmARF29-F	ATGATGGCCTCCTCGCAGGAGAA
	GRMZM2G086949	ZmARF29-R	AGCTATTTGGATGCAGTCGTTCAAG
	GRMZM2G475882	ZmARF30-F	ATGAGCTCCTCGTCCGCG
	GRMZM2G475882	ZmARF30-R	AGATAGGTAGCGTGGATCGTT
	GRMZM2G181254	ZmARF32-F	CGAGAAGAGCCGAAGAGGGC
	GRMZM2G181254	ZmARF32-R	CCTTCGTGCTTCGAGTTGCGT
	GRMZM2G179121	ZmARF33-F	ATGGCGACGCAGCAACCG
	GRMZM2G179121	ZmARF33-R	GGAGGAGGGAGGAGCTCG
	GRMZM2G081158	ZmARF34-F	ATGAACCTCTCCCCGCCC
	GRMZM2G081158	ZmARF34-R	GTAGTCCAGAGGCACCAC

Purpose	Gene ID	Name	Sequence (5'-3')
qRT auxin related genes	GRMZM2G156470	SAUR2-F	TGCCTTAGCACCCCTGTCTT
	GRMZM2G156470	SAUR2-R	AGGCTCCTCTCCTGAGCAAA
	GRMZM2G042741	SAUR40-F	TGCGGAGGAGTTTGGTTTCA
	GRMZM2G042741	SAUR40-R	AGCTTGATTGCTGACAAGGC
	GRMZM2G042429	SAUR52-F	TCAGGGAGCTCCTGCAAATG
	GRMZM2G042429	SAUR52-R	TGAGCAAGCACATGGCGTA
	GRMZM2G156451	SAUR61-F	GTACGCCATGTGTTTGCTCA
	GRMZM2G156451	SAUR61-R	AATCTGCTGTCTGGCTCGTG
	GRMZM2G079957	IAA2-F	GGGTGTTCGTGAAGGTGAGC
	GRMZM2G079957	IAA2-R	GAGAAGCAGAGGAACATGGCT
	GRMZM5G825707	IAA6-F	CATGCCGGTGATTTGACAGC
	GRMZM5G825707	IAA6-R	CGTCGCTCGACATGTAGGTA
	GRMZM5G864847	IAA20-F	CCGGCTACGACACGCTAA
	GRMZM5G864847	IAA20-R	TCGTCGTCCTCGTACACCA
	GRMZM2G098643	PIN1-F	GCGCAAACTCATCCGTAACC
	GRMZM2G098643	PIN1-R	TGGACTTGAGGATGATCGCC
	GRMZM2G025742	PIN5-F	AACCCCAACACGTACGCC
	GRMZM2G025742	PIN5-R	GCCCGACTTGGACATGATGAG
	GRMZM5G859099	PIN9-F	TCCTTGGCCTCATCTGGTCT
	GRMZM5G859099	PIN9-R	GCGCTATGAACGTCCCTGAA
	GRMZM2G129413	AIC1-F	ACAGGCGCTTACGTCATCAA
	GRMZM2G129413	AIC1-R	CACTGGTAGCACTTGGCGA
	GRMZM2G045057	AIC2-F	CACGCCACTCTACTTCGTGT
	GRMZM2G045057	AIC2-R	GTTGATGGGGCCGAAAAACG
qRT cell cycle related genes	GRMZM2G178215	CYCA3-1-F	TCCACCTTGCGGTTTCCTAC
	GRMZM2G178215	CYCA3-1-R	GTCTCCATCTTGACCACTTGC
	GRMZM2G061287	CYCB2-4-F	CAGCAGACGCAGATAAACAGC
	GRMZM2G061287	CYCB2-4-R	GGAGTTGGTCGCCAGTGTAT
	GRMZM2G050933	CYCD6-F	CTTCTTCCTCTCCGCATGCTAT
	GRMZM2G050933	CYCD6-R	GAGAACTCCGCCATCTTCAC
	GRMZM2G073003	CYC7-F	AGCTTGTCGGCGTTTCATCT
	GRMZM2G073003	CYC7-R	GACAGTCAGGTTCCACTCCAG
	GRMZM2G138886	CYC8-F	ATCTGACCGTGCCTACACAA
	GRMZM2G138886	CYC8-R	TACCAAGCAGAGCTCCAGCA
	GRMZM2G006721	CYC15-F	ACCCTCGAGTGGCGGAT
	GRMZM2G006721	CYC15-R	GGATGCTACGGCGATGGTC
	GRMZM2G008327	CDKB2-F	TTGGAGCCACGCACTACTC
	GRMZM2G008327	CDKB2-R	GCCACACCTGTTCGTTTGG
	GRMZM2G084347	CDK12-F	CGCTTAACAGCGTCTTCTGC
	GRMZM2G084347	CDK12-R	CGTGTTCTCGTCTTCTTGGC
qRT cell expansion related genes	GRMZM5G886185	XTH12-F	CCCATGTACGCCTACTCCAG
	GRMZM5G886185	XTH12-R	GGTCGATGCCGTGGTAGTT
	GRMZM2G081790	EXT-F	AGTTCCGGATGTGGTTCGAC
	GRMZM2G081790	EXT-R	CCACACGCTGGCGTACA
	GRMZM2G048474	PER-F	TCGCCAAGATCGTCACCTTC
	GRMZM2G048474	PER-R	CTTGTTCCTGATCTCGCCCG
	GRMZM2G070603	PER1-F	CGCACTGCCCGTCGTA
	GRMZM2G070603	PER1-R	AGCTCGTGTCGAAGGTCTTG
	GRMZM2G451097	PERL2-F	CCGACTTCCTGGACAACTCC
	GRMZM2G451097	PERL2-R	AAGTCCTCGTCCCATAGCGT
	GRMZM2G149273	PER5-F	TCGGCAACTTCGGTAACAGG
	GRMZM2G149273	PER5-R	TCTTCTCCTTGTCTCCGTTGC
	GRMZM2G048474	PRX47-F	GTCTCGTGCGCGGACATC
	GRMZM2G048474	PRX47-R	CCTCTCGAGGCTGGCGAA

Purpose	Gene ID	Name	Sequence (5'-3')
Mutator Mutant confirmation	None	TIR8.1	CGCCTCCATTTCGTCGAATCCCCTS
	None	TIR8.2	CGCCTCCATTTCGTCGAATCCSCTT
	None	TIR8.3	SGCCTCCATTTCGTCGAATCCCKT
	None	TIR8.4	CGCCTCCATTTCGTCGAATCACCTC
	GRMZM2G085113	ZmTE1-F1	TCGTGGTGCTGGGCCTTGT
	GRMZM2G085113	ZmTE1-R1	TTCAGCCGCCCACAGGGTGATG
EMS mutant confirmation	GRMZM2G085113	ZmTE1-F2	TCGTGGTGCTGGGCCTTGT
	GRMZM2G085113	ZmTE1-R1	TTCAGCCGCCCACAGGGTGATG
	GRMZM2G324157	GRMZM2G324157-F	TCTACGCCGCCGCTGCGC
	GRMZM2G324157	GRMZM2G324157-R	CTACGCTACGATTGCCTCGC
	GRMZM2G124911	GRMZM2G124911-F	ATGGGCTCCGGGGAGGAGC
	GRMZM2G124911	GRMZM2G124911-R	TCACATCAGTATCGACTCTAA
	GRMZM2G039650	GRMZM2G039650-F	CCCTTCTCCGCCTTCATT
	GRMZM2G039650	GRMZM2G039650-R	TCAGCCATTGCATCTTAC
In situ hybridization probe	GRMZM2G085113	ZmTE1-F3	AAAGGACGAAAGAGCGGGTGG
	GRMZM2G085113	ZmTE1-R3	GCGGCTTGGCTACGACGAC
	GRMZM2G017087	ZmKN1-F	CAAAGAAGAAGAAGAAAGGGAA
	GRMZM2G017087	ZmKN1-R	CAGGTTACGCATACAATACGAC
BiFC	GRMZM2G085113	ZmTE1-F	ATGGAGGGTGGGGGAGGGAG
	GRMZM2G085113	ZmTE1-R	GTCGTCGTAGCCAAGCCG
	GRMZM5G878541	ZmWEE1-F	ATGCCCCCGCCGCCGCCG
	GRMZM5G878541	ZmWEE1-R	ATGCCCCCGCCGCCGCCG
	GRMZM2G108355	ZmPP2A-F	ATGAGCAGCCCGCATGGC
	GRMZM2G108355	ZmPP2A-R	GACACCTGACTATTTCCTGTGA
CoIP	GRMZM2G085113	ZmTE1-F	ATGGAGGGTGGGGGAGGGAG
	GRMZM2G085113	ZmTE1-R	GTCGTCGTAGCCAAGCCG
	GRMZM5G878541	ZmWEE1-F	ATGCCCCCGCCGCCGCCG
	GRMZM5G878541	ZmWEE1-R	ATGCCCCCGCCGCCGCCG
	GRMZM2G108355	ZmPP2A-F	ATGAGCAGCCCGCATGGC
	GRMZM2G108355	ZmPP2A-R	GACACCTGACTATTTCCTGTGA
Subcelluar localization	GRMZM2G085113	ZmTE1-F	ATGGAGGGTGGGGGAGGGAG
	GRMZM2G085113	ZmTE1-R	GTCGTCGTAGCCAAGCCG
	GRMZM5G878541	ZmWEE1-F	ATGCCCCCGCCGCCGCCG
	GRMZM5G878541	ZmWEE1-R	ATGCCCCCGCCGCCGCCG
	GRMZM2G108355	ZmPP2A-F	ATGAGCAGCCCGCATGGC
	GRMZM2G108355	ZmPP2A-R	GACACCTGACTATTTCCTGTGA
qRT Control gene	GRMZM2G046804	ZmGAPDH-F	GTCTCGTCCACCCGTCATCCT
	GRMZM2G046804	ZmGAPDH-R	CCTCCAATGGATCCTCGTTA
	GRMZM2G126010	ZmACTIN1F	GAGCGGGAGATTGTCAGGG
	GRMZM2G126010	ZmACTIN1R	AAGGGATGGTTGGAACAGCA
qRT	GRMZM2G085113	Q-ZmTE1-F	ATCTGCCAAGTGACATACGC
	GRMZM2G085113	Q-ZmTE1-R	GTACTCGTCGCTGTCGCAC
